# Editorial: Reducing health disparities: promoting vulnerable older adults' psychological health

**DOI:** 10.3389/fpsyg.2023.1187403

**Published:** 2023-04-20

**Authors:** Yufang Guo, Anni Wang, Ruishuang Zheng

**Affiliations:** ^1^School of Nursing and Rehabilitation, Shandong University, Jinan, China; ^2^School of Nursing, Fudan University, Shanghai, China; ^3^Department of Hepatobiliary Cancer, Tianjin Medical University Cancer Hospital and Institute, National Clinical Research Centre for Cancer, Tianjin, China

**Keywords:** psychological health, older people, health promotion, leisure activities, resilience, psychological capital

With a rapidly aging population worldwide, more people are expected to live to their 60s and beyond. Since 2019, the population of adults aged 60 and above have exceeded 1 billion, and it is projected to exceed 1.4 billion by 2030 and 2.1 billion by 2050 (WHO, 2018). Given the rapid increase in the older adult population, mental health disorders have become a critical issue and affect a sizable portion of older adults, especially after the wide spread of the Coronavirus disease 2019 (COVID-19) (Segal et al., 2018). Studies show that 10 to 50% of older adults experience mental health problems (e.g., anxiety, depression, and stress-related mental health disorders) and 24 to 46% of them report their mental health issues are positively associated with COVID-19-related worries, social distancing, physical distancing, isolation (Kim and Ko, 2018; Maral and Punetha, 2022). Compared with younger cohorts, older adults with mental health problems tend to have worse emotional regulation and cognitive function, which are frequently comorbid with sleep disturbance, loneliness, disability, physical illnesses, worse quality of life and higher risk of suicide (Kastenschmidt and Kennedy, 2011; Grossman et al., 2020). Therefore, mental health problem detection and referral for older adults should be an important concern for health administrators, educators, caregivers and researchers. This Research Topic, “*Reducing health disparities: promoting vulnerable older adults' psychological health,”* aims to collect and present researchers' studies on evaluating, preventing and managing mental health problems among older adults.

This Research Topic was presented to Frontiers in Psychology (Psychology of Aging). From the 28 June 2022 to 28 December 2022, four manuscripts were published, including a systematic review (Yang et al.), a quantitative study (Zhang Y. et al.), a measurement translation (Zhang X. et al.) and a measurement development and investigation (Xin and Li).

Several studies have reported that physical, social, intellectual, artistic and cultural activities are correlated with age-related changes in cognitive abilities (Andel et al., 2016; Mella et al., 2017). Yang et al. employed a systematic review and meta-analysis to explore the effect of leisure activities on cognitive aging among older adults. Nineteen prospective cohort studies were included, and among them, 8 studies were conducted in European and American countries, and eleven studies were conducted in Asian countries. Meta-analysis showed that the positive effects of leisure activities on dementia (pooled RR 0.80), cognitive impairment risk (pooled RR 0.67) and cognitive decline risk (pooled RR 0.87) were significant among the older population.

For the community-dwelling older population, Zhang Y. et al. found that the prevalence of mild cognitive impairment was higher in older adults who took family-centered activities than those who took multidomain activities and self-improvement activities. Through a prospective study, Ren et al. (2022) reported that participation in leisure activities (including keeping domestic animals or pets, taking part in social activities, reading books or newspapers and playing cards, or mahjong) was moderately associated with an increase in cognitive function. An analysis from a national survey including 23,694 older adults also supported that leisure activities functioned as a protective factor against cognitive impairment without dementia and dementia (Guerrero Barragan et al., 2021). Therefore, further studies are needed to clarify the relationships between types of leisure activities and cognitive decline in older individuals.

Recently, personal intrinsic capacity has attracted increased interest among researchers and is believed to be an important factor for alleviating age-related mental and physical problems (Chhetri et al., 2021). Zhang X. et al. developed a Chinese version of the Walsh Family Resilience Questionnaire for community-dwelling disabled elderly individuals, which had good reliability and validity. Xin and Li proposed the structure of psychological capital for older adults. They found that psychological capital of older adults comprised resilience, self-efficacy, optimism, ease and content, gratitude and dedication, wisdom, and meaning in life. Based on the content of psychological capital, they developed a psychological capital questionnaire for older adults. Then, an investigation was conducted and negative correlations between psychological capital, its seven factors and depression among older adults was found, which indicated that psychological capital significantly impacted depression of older adults. Previous studies have indicated that trait resilience and psychological capital are important resources for maintaining mental health and mitigating cognitive impairment (Farber and Rosendahl, 2020). These studies emphasized the necessity for developing psychological interventions for older adults to improve their resilience, psychological capital and other positive characteristics (Clark et al., 2019). For example, Bartholomaeus et al. (2019) developed a community intervention to promote wellbeing, resilience, optimism and social connection for older adults and their caregivers.

## For the future

Concerns about mental health crises among older populations began to increase worldwide during the post-COVID-19 era. The mental health problems presented in this Research Topic are just the tip of the iceberg. It is necessary to investigate the mental health status of older adults, identify the influencing factors (e.g., personal characteristics, family-related factors, social, and cultural factors) and investigate the pattern of influencing factors on mental health problems. Moreover, effective psychological and physical interventions, such as mindfulness therapy, meditation, exercise and virtual reality exergames, should be promoted and applied in older populations (Beauchamp et al., 2021; Murfield et al., 2021). Finally, considering the differences in ethnicity, culture, social structure, population and economy among countries, the roles of governments and health administration institutes in promoting the mental health of older adults should be explored in greater detail.

## Author contributions

YG completed the first version of the editorial. AW and RZ revised the editorial. All authors approved the submitted version of this editorial.
